# Computed Tomography (CT) Perfusion as an Early Predictive Marker for Treatment Response to Neoadjuvant Chemotherapy in Gastroesophageal Junction Cancer and Gastric Cancer - A Prospective Study

**DOI:** 10.1371/journal.pone.0097605

**Published:** 2014-05-20

**Authors:** Martin Lundsgaard Hansen, Eva Fallentin, Carsten Lauridsen, Ian Law, Birgitte Federspiel, Lene Bæksgaard, Lars Bo Svendsen, Michael Bachmann Nielsen

**Affiliations:** 1 Department of Radiology, Rigshospitalet, Copenhagen, Denmark; 2 Bachelor’s Degree Programme in Radiography, Department of Technology, Metropolitan University College, Copenhagen, Denmark; 3 Department of Clinical Physiology, Nuclear Medicine and PET, Rigshospitalet, Copenhagen, Denmark; 4 Department of Pathology, Rigshospitalet, Copenhagen, Denmark; 5 Department of Oncology, Rigshospitalet, Copenhagen, Denmark; 6 Department of Surgical Gastroenterology, Rigshospitalet, Copenhagen, Denmark; Stanford University School of Medicine, United States of America

## Abstract

**Objectives:**

To evaluate whether early reductions in CT perfusion parameters predict response to pre-operative chemotherapy prior to surgery for gastroesophageal junction (GEJ) and gastric cancer.

**Materials and Methods:**

Twenty-eight patients with adenocarcinoma of the gastro-esophageal junction (GEJ) and stomach were included. Patients received three series of chemotherapy before surgery, each consisting of a 3-week cycle of intravenous epirubicin, cisplatin or oxaliplatin, concomitant with capecitabine peroral. The patients were evaluated with a CT perfusion scan prior to, after the first series of, and after three series of chemotherapy. The CT perfusion scans were performed using a 320-detector row scanner. Tumour volume and perfusion parameters (arterial flow, blood volume and permeability) were computed on a dedicated workstation with a consensus between two radiologists. Response to chemotherapy was evaluated by two measures. Clinical response was defined as a tumour size reduction of more than 50%. Histological response was evaluated based on residual tumour cells in the surgical specimen using the standardized Mandard Score 1 to 5, in which values of 1 and 2 were classified as responders, and 3 to 5 were classified as nonresponders.

**Results:**

A decrease in tumour permeability after one series of chemotherapy was positively correlated with clinical response after three series of chemotherapy. Significant changes in permeability and tumour volume were apparent after three series of chemotherapy in both clinical and histological responders. A cut-off value of more than 25% reduction in tumour permeability yielded a sensitivity of 69% and a specificity of 58% for predicting clinical response.

**Conclusion:**

Early decrease in permeability is correlated with the likelihood of clinical response to pre-operative chemotherapy in GEJ and gastric cancer. As a single diagnostic test, CT Perfusion only has moderate sensitivity and specificity in response assessment of pre-operative chemotherapy making it insufficient for clinical decision purposes.

## Introduction

Computed Tomography (CT) perfusion can visualise changes in tumours vascular physiology, and could thereby potentially provide a biomarker for therapeutic response to chemotherapy [1]–[8]. With the advent of multidetector row CT scanners and improved image reconstruction methods, larger structures and entire organs can be analysed in a single CT perfusion study. Potentially, CT perfusion could supplement CT or PET/CT imaging as a one-stop modality for cancer staging and therapy assessment in cancer imaging.

In patients with locally advanced esophageal cancer, gastroesophageal junction (GEJ) cancer and gastric cancer perioperative chemotherapy improves overall and progression-free survival as confirmed by several randomised trials and meta-analyses [9]–[11]. Accordingly, a regimen of 9 weeks of chemotherapy prior to surgery is now included in the Danish national guidelines and is a standard treatment in our institution. The individual response to chemotherapy varies between 30 and 60% depending on evaluation methods [12]–[15] based either on histological or clinical response. The consequence to patients not responding to treatment is a delay in their definitive surgical treatment. Moreover, it is important to identify non-responders, as continuous chemotherapy carries a risk of on-going progression, non-curability, side effects to chemotherapy and costs related to ineffective chemotherapy. To optimise and individualise treatment an imaging modality predicting or assessing treatment response at an early time point after treatment start would, thus, be of great value.

Our hypothesis was that a reduction in perfusion parameters could predict treatment response of pre-operative chemotherapy. To address this hypothesis changes in CT perfusion parameters in GEJ and gastric cancer during preoperative chemotherapy were measured and analysed for correlation with both clinical and histological response.

## Materials and Methods

### Patients

From October 2011 to January 2013 thirty consecutively admitted patients with biopsy confirmed adenocarcinoma at the gastro-esophageal junction or in the stomach were included in the study after being considered potentially resectable at a multidisciplinary tumour (MDT) conference and offered preoperative chemotherapy. Exclusion criteria were: allergy to contrast material, impaired renal function, and patient unfitness for chemotherapy. Of these thirty patients, one patient completed only the first scan and one patient was excluded from the study due to motion artefacts appearing in all three perfusion scans. The remaining 28 patients constituted the study group (24 males, 4 females, median age 65, range 44–79). Twenty-six patients underwent all three scans as described below. One patient was excluded from the third scan due to elevated serum creatinine levels, and one patient did not undergo the second scan due to hardware failure, leaving 27 patients for evaluation after one series of chemotherapy and 27 for presurgical responses. The diagnostic workup consisted of an upper endoscopy with biopsy, CT of the chest and abdomen combined with ultrasound of the neck and/or PET-CT for tumour staging, and after MDT conference a diagnostic laparoscopy to rule out peritoneal carcinomatosis. Patients with locally advanced tumour (T2– T4), with or without lymph node involvement (N0– N3) and no distant metastases (M0) were included. Methods and results are presented according to the REMARK recommendations for biomarker studies [16] - see supplementary Checklist S1.

### Ethics

The research protocol was approved by the Committees on Biomedical Research for the Capital Region of Denmark (protocol number H-1-2010-132). All patients gave oral and written informed consent according to the Helsinki II Declaration.

### Perfusion CT

All CT perfusion examinations were performed using a 320-detector row CT scanner (Aquilion ONE, Toshiba Medical Systems, Ohtawara, Japan). The study protocol consisted of three perfusion scans: baseline (median 8 days before starting chemotherapy, range 3–37 days), after the first cycle of chemotherapy (median 20 days, range 18–31 days) and - before surgery - after three cycles of chemotherapy (median 79 days, range 61–102 days). The last scan was performed at a median of 6 days before surgery (range 1–21 days).

Each patient was instructed to fast 2-hours prior to the CT examination, and was given 500 mL of water just prior to the CT perfusion study to distend the stomach for better visualisation of the tumour. The anticholinergic, hyoscine butylbromide, was administered (20 mg, *I.V.*) as a gastrointestinal motility inhibitor. An abdominal strap was placed around the patient to reduce movement and to encourage shallow breathing during the examination.

An area of 12–16 cm which covered the extent of the tumour was selected, and this area was used for all subsequent scans. A 16–18-gauge catheter was placed in the antecubital vein for administration of contrast material. Twenty mL of non-ionic contrast material (Omnipaque 350; GE Healthcare) was used as a test-bolus (single slice acquisition, 1-second intervals for 20 seconds) to determine the initial delay from contrast administration to arrival in the aorta. The arrival of contrast was visually determined and the delay for the CT perfusion scan was set to assure the acquisition of 2 full scan volumes without contrast material in the dynamic sequence.

The CT perfusion scan protocol consisted of 19 consecutive volumes divided into 3 phases: The first phase consisted of 11 volumes at 2 second intervals, a second phase of 6 volumes at 3 second intervals, and a third phase of 2 volumes at 5 second intervals. The first volume scan started between 7.5 and 13.5 seconds after contrast administration, and the total acquisition time ranged from 55 to 60 seconds. The contrast volume ranged from 30 to 40 mL, dependant on patient bodyweight (below 50 kg: 30 mL, between 50 and 79 kg: 35 mL, above 80 kg: 40 mL) followed by a saline flush of 30 mL. The injection rate varied from 5 to 8 mL/s, so that the overall contrast injection time never exceeded 5 seconds. The image acquisition used the following parameters: 100 kV, 100 mA, 0.5 s/rotation time, fixed table position, and 0.5 mm reconstruction.

### Image Post Processing and Analysis

Images were reconstructed using AIDR 3D (Adaptive Iterative Dose Reduction, Toshiba Medical Systems, Japan). All datasets were corrected for motion using a non-rigid registration algorithm on the scan console (Toshiba). The volume containing the peak aortic enhancement was selected as a reference phase for motion correction. In two cases, the two late volumes were scanned at a different table position, and could therefore not be included in the motion correction.

The reconstructed, motion-corrected datasets were transferred to a stand-alone workstation (Vitrea 6.3, Vital Images, Toshiba Medical Systems, Minnetonka, USA) for post-processing. For data analysis, the input artery was selected by placing a 100 mm^2^ circular Region of Interest (ROI) in the centre of the abdominal aorta and a second ROI was placed in the tumour. Parametric perfusion maps were generated with the following perfusion parameters: Arterial flow (tissue perfusion measured in mL⋅min^−1^⋅100 g^−1^), blood volume (measured in mL⋅100 g^−1^) and permeability (measured as k^trans^ in mL⋅min^−1^⋅100 g^−1^). Based upon consensus between two radiologists, a free-hand ROI was drawn around the tumour-border on slices containing visible tumour and these circumscribed areas were utilized to define tumour volume using a sculpt-tool. Care was taken to exclude esophageal and gastric lumen and surrounding tissue. A ROI was also drawn on baseline scans in healthy appearing gastric tissue.

### Treatment

Patients were Scheduled for Three Cycles of Chemotherapy Preoperatively. after Completion of the Study, Patients Were Scheduled for Postoperative Chemotherapy, in Accordance with the MAGIC Study [10]. Each 3-Week Cycle Consisted of Epirubicin (50 Mg per Square Meter of Body-Surface Area) Intravenously on Day 1, Cisplatin (60 Mg per Square Meter) Or Oxaliplatin (130 Mg per Square Meter on Day 1) Intravenously on Day 1, Combined with Peroral Capecitabine (500 Mg per Square Meter Twice Daily) Continuously for 21 Days. Substituting Cisplatin with Oxaliplatin Has Proven to Be Non-Inferior and Was Considered an Alternative Regimen [17]. before and during Each Cycle of Treatment, a Full Blood Analysis Was Obtained Including Serum Creatinine Level. If Myelosuppression Or Thrombocytopenia Was Observed, Cycle 2 Or Cycle 3 Was Postponed until Normalisation of the Blood Count. Patients Were Closely Monitored for Side Effects during Chemotherapy and Modifications of the Dose Were Made Based on Observed Toxicity. Treatment Changes and Decisions Were Made Blinded for Any Changes Measured Using CT Perfusion. One Patient Did Not Start the Third Series of Chemotherapy Due to Side Effects. This Patient Was Later Classified as Both a Clinical and a Histological Responder.

### Surgery

In patients with tumour at the gastro-esophageal junction an Ivor-Lewis Esophagectomy with extended D1+ lymphadenectomy was performed. In patients with a stomach tumour a complete gastrectomy was performed. One patient (tumour at the gastroesophageal junction) was not resected due to local tumour growth invading the pancreas and was because of this classified as a clinical nonresponder without histological grading.

### Clinical and Histological Response Evaluation

**Figure 1:**
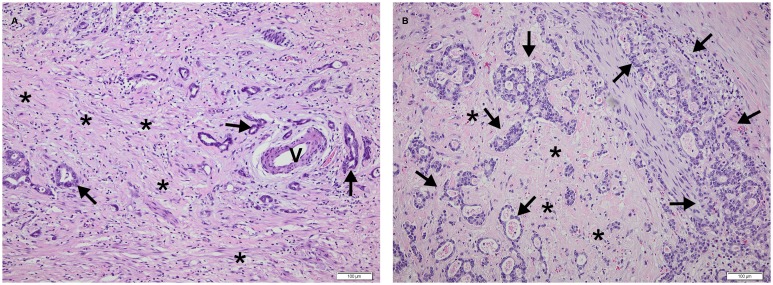
Illustration of histological response evaluation using Mandard score. A) Tumour regression grade 2 (Mandard). Few, scattered residual cancer areas (arrows) separated by fibrosis (*). One tumour area is surrounding a vessel (V) and another tumour area is seen to the left. This patient was a histological responder. B) Tumour regression grade 4 (Mandard). Residual cancer areas (arrows) outgrowing fibrosis (*). This patient was a histological nonresponder.

Responses were evaluated both clinically and histologically. Investigators performing the evaluations were blind to patient outcome and results of the imaging studies. For the clinical response evaluation, the endoscopic imaging and tumour length, measured with a straight endoscope at the level of the incisors, before chemotherapy was compared with the macroscopic measurements of tumour in the surgical specimen – taking tumour shrinkage of 10% due to fixation into account [18]. Patients with a tumour length reduction of more than 50% were classified as clinical responders. For the histological response evaluation, all surgical resection specimens were evaluated by a single experienced pathologist and the tumour response was graded according to a score established by Mandard et al [19]. Patients with no or a few scattered small tumour areas (regression score 1 and 2) were classified as responders. Patients with larger residual tumour areas (regression score 3,4 and 5) were classified as non-responders – see Figure 1A and 1B.

Tumour perfusion parameters at baseline were compared with perfusion parameters from normal gastric tissue. Changes between tumour perfusion parameters at baseline and the second, and third scans, were calculated using the following formulas, respectively, where brackets represent a perfusion measure:
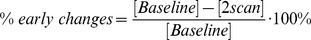


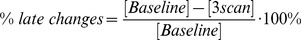



### Statistics

All statistical tests were performed using SPSS (version 20, IBM, USA). Perfusion parameters at baseline and at the third scan were compared between responders and nonresponders using Mann-Whitney U-test. Changes in tumour perfusion between first and second scans, and changes between first and third scans were compared between groups based on response type using univariate logistic regression. Comparisons of tumour tissue perfusion and normal gastric tissue perfusion was performed by using a Wilcoxon signed-rank test. A 2-sided p-value below 0.05 was considered statistically significant. A Receiver Operating Characteristic (ROC) analysis was made to determine the optimal diagnostic cut–off value for changes in perfusion parameters in regards of sensitivity and specificity for predicting clinical response.

## Results

### Baseline Values, Clinical and Histological Response

Of the 28 patients available for clinical response evaluation, 13 were classified as clinical responders and 15 were classified as nonresponders. One patient was not resected due to local tumour growth invading the pancreas and was because of this classified as a clinical nonresponder. Based on histological examination, eight cases (Mandard score 1 and 2, complete or subtotal regression) were classified as responders and 19 cases (Mandard score 3, 4 and 5) were classified as nonresponders. Table 1 summarises statistical results of baseline scanning of clinical responders and nonresponders. There were no significant statistical differences between the two groups at baseline. With regard to the histological response evaluation, the responding patients had significantly smaller tumours (p<0.05). Table 2 summarises differences in clinical and histological response evaluation. Comparison of tumour tissue perfusion and perfusion measured in normal gastric tissue revealed a significantly higher perfusion in tumour versus gastric tissue (116.0 mL⋅min^−1^⋅100 g^−1^ versus 77.2 mL⋅min^−1^⋅100 g^−1^, p<0.01).

**Table 1:** Baseline perfusion parameters and tumour volume.

	Clinical Nonresponders (n = 15)	Clinical responders (n = 13)	p-value
**Arterial flow**	107.4 (96.5–146.0)	116.1 (84.5–140.8)	p = 0.79
mL⋅min^−1^⋅100g^−1^			
**Blood volume**	6.7 (4.1–7.5)	6.3 (3.4–9.6)	p = 0.90
mL⋅100g^−1^			
**Permeability (k^trans^)**	25.2 (20.9–34.9)	28.9 (23.0–30.3)	p = 0.79
mL⋅min^−1^⋅100g^−1^			
**Tumour volume**	34.3 (22.7–59.9)	30.8 (9.8–51.6)	p = 0.25
mL			

Meassurements for clinical responders and non-responders. Values are medians and interquartile range in parentheses.

**Table 2:** Clinical versus histological response.

	Clinical Nonresponders (n = 15)	Clinical Responders (n = 13)
***Histological response (n = 27)***		
Mandard 1		4
Mandard 2	1	3
Mandard 3	1	3
Mandard 4	8	2
Mandard 5	4	1
Not resected	1	
***Tumour t-staging at baseline***		
≤T2	1	5
= T3	12	7
= T4	2	1

Clinical response compared to histological response (Mandard Score 1 to 5) and tumour stage based on CT evaluation before chemotherapy.

### Clinical Response: Changes in Perfusion Parameters and Tumour Volume after First Series of Chemotherapy

**Figure 2:**
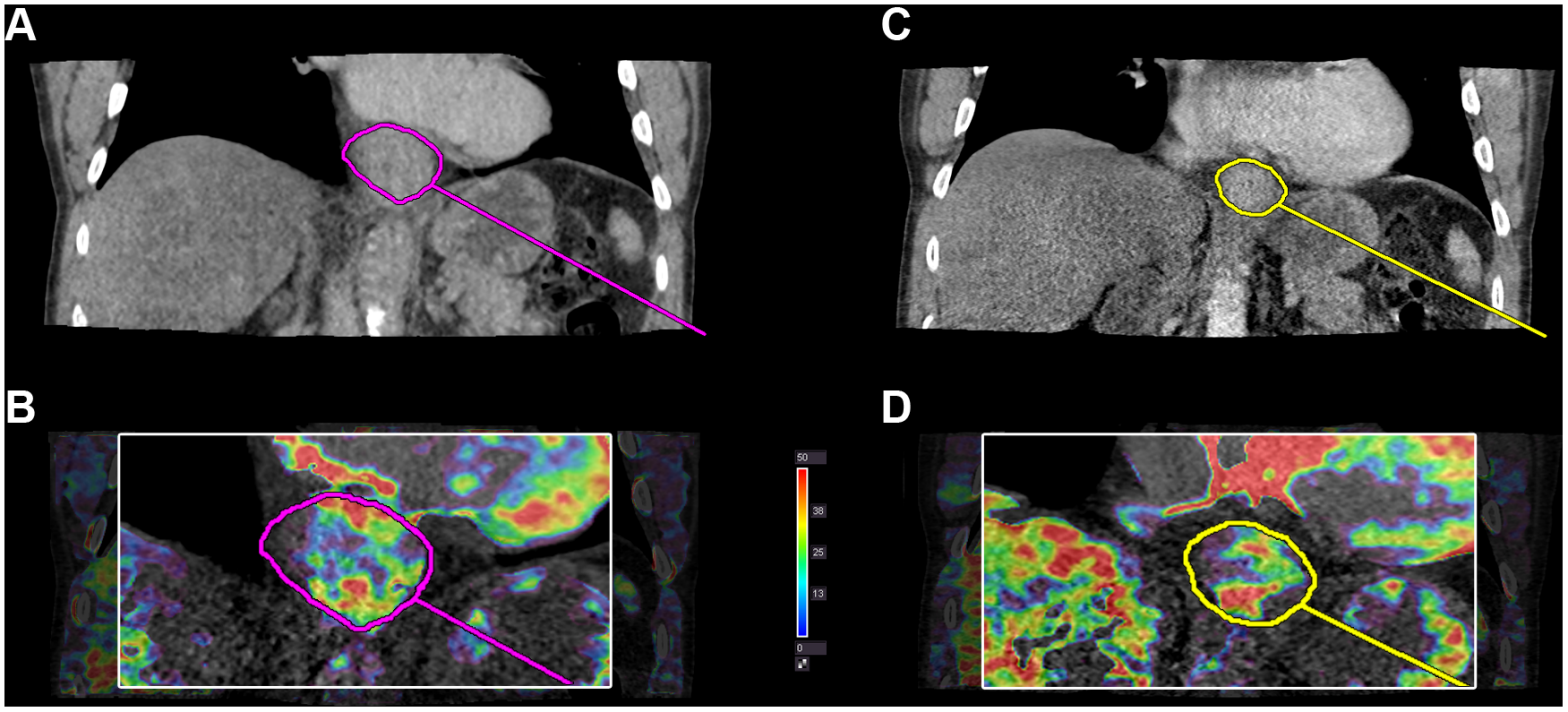
CT perfusion images of a clinical responder. Permeability k^trans^ parametric map in a clinical responder with a reduction in permeability between baseline and the first series of chemotherapy. **A+B)** Before chemotherapy (k^trans^ = 32.1 mL⋅min^−1^⋅100 g^−1^) **C+D)** After first series of chemotherapy (k^trans^ = 23.9 mL⋅min^−1^⋅100 g^−1^). Perfusion measures are averages from the entire tumor volume. The image illustrates perfusion measured in a reconstructed coronal plane which is possible with volume perfusion.

**Figure 3:**
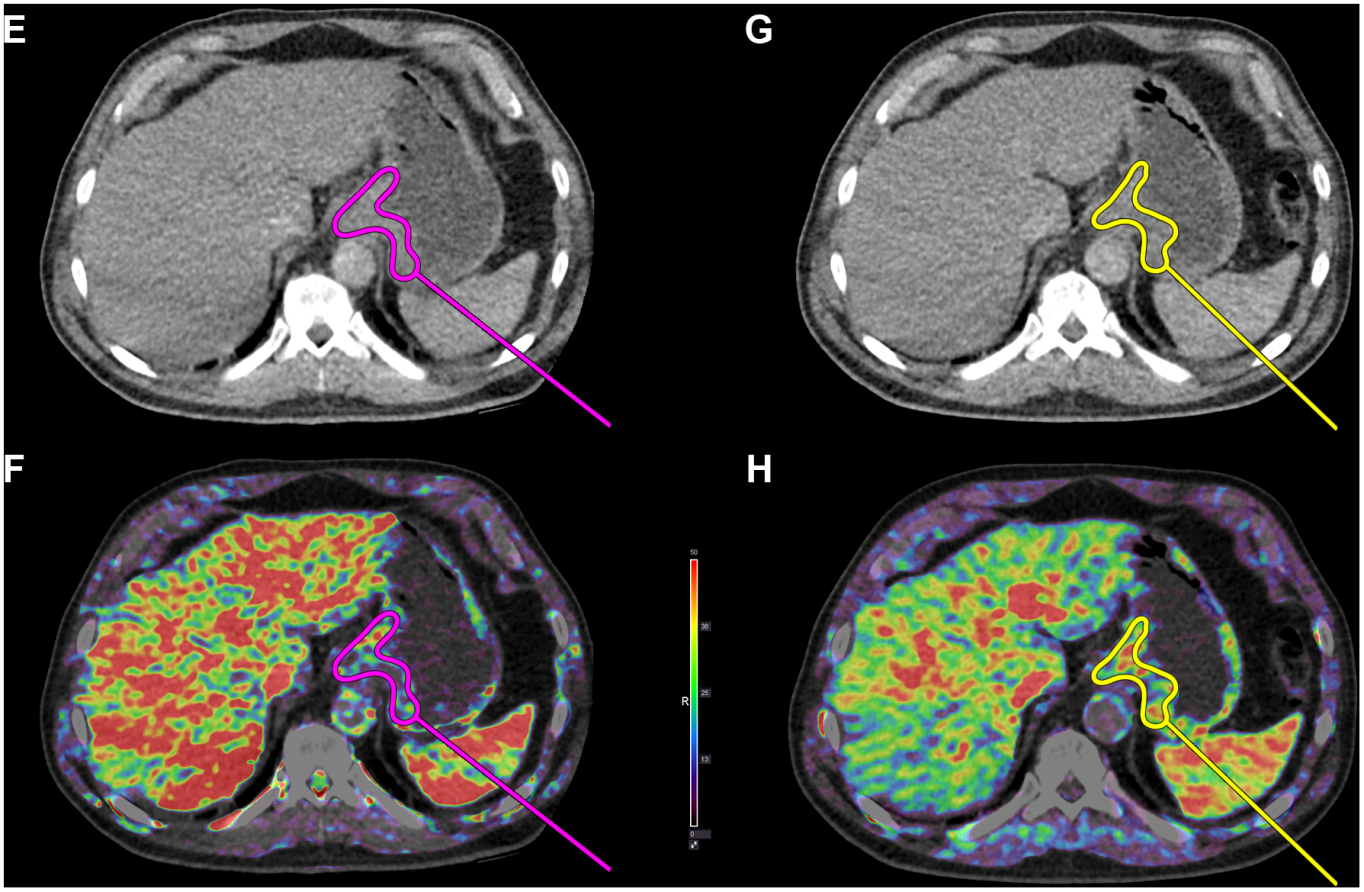
CT Perfusion images of a clinical nonresponder. Permeability k^trans^ in a clinical nonresponder. An increase in k^trans^ is observed. **E+F)** Before chemotherapy (k^trans^ = 13.7 mL⋅min^−1^⋅100 g^−1^) and **G+H)** After first series of chemotherapy (k^trans^ = 21.0 mL⋅min^−1^⋅100 g^−1^). Perfusion measures are averages from the entire tumor volume.

**Figure 4:**
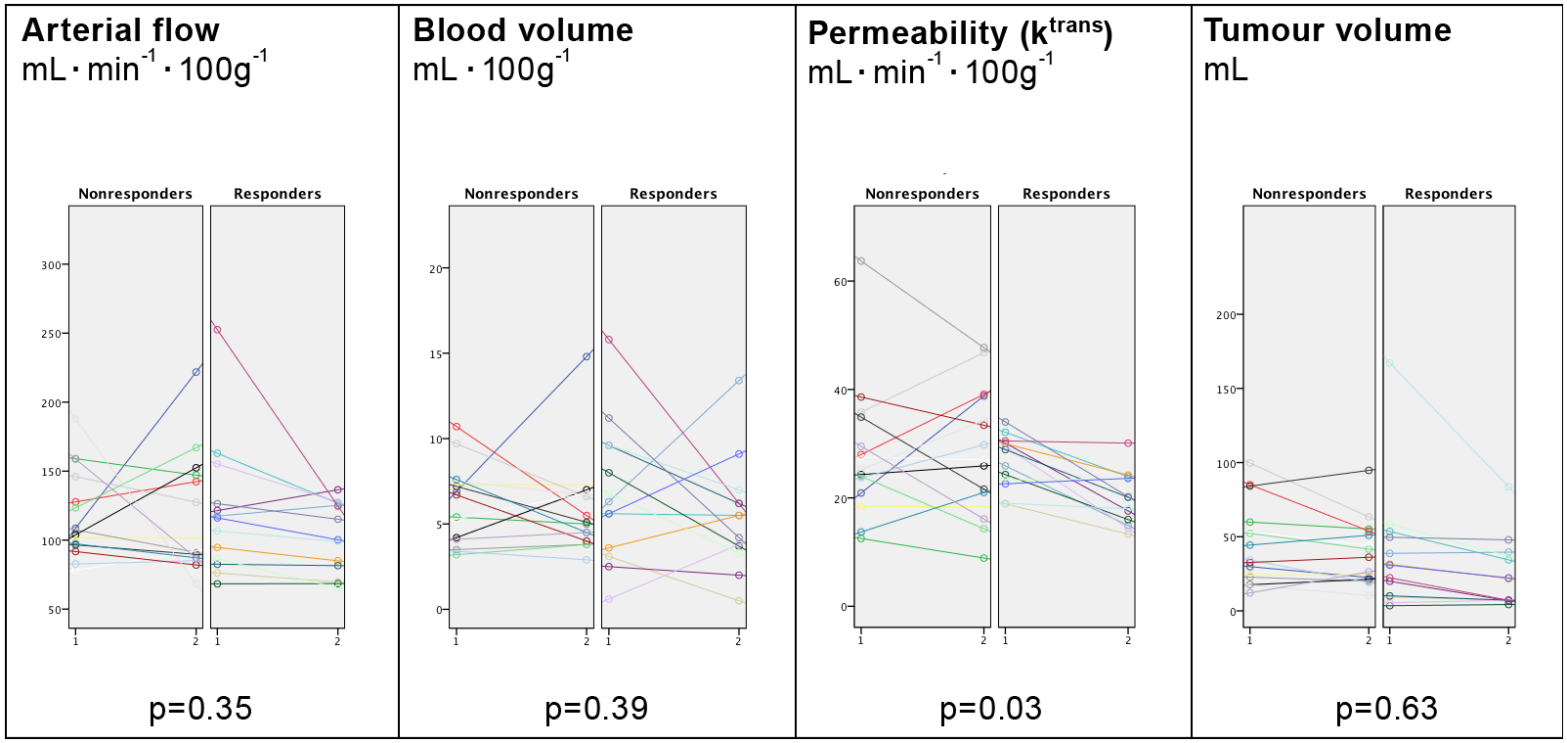
Early changes in perfusion parameters and tumour volume in clinical responders. Changes from baseline to first follow-up scan after first series of chemotherapy for the 27 cases available for early follow up evaluation. Each case is illustrated with a line between the baseline and the second scan. All values are in absolute numbers.

Figure 2 and 3 illustrate a clinical responder and a clinical nonresponder. Figure 4 illustrates a comparison of changes in tumour size and perfusion parameters from baseline to the first series of chemotherapy based on clinical response. A logistic regression analysis showed that response probability was positively correlated to a decrease in tumour permeability (p = 0.03). Eighteen out of 27 (67%) patients had decreases in permeability between baseline and after the first series of chemotherapy, whereof 11/13 (85%) were clinical responders and 7/14 (50%) were clinical nonresponders. This difference was not statistically significant (p = 0.10, Fisher’s exact test). There were no significant changes in arterial flow, blood volume or tumour volume size when comparing responders to nonresponders.

**Figure 5:**
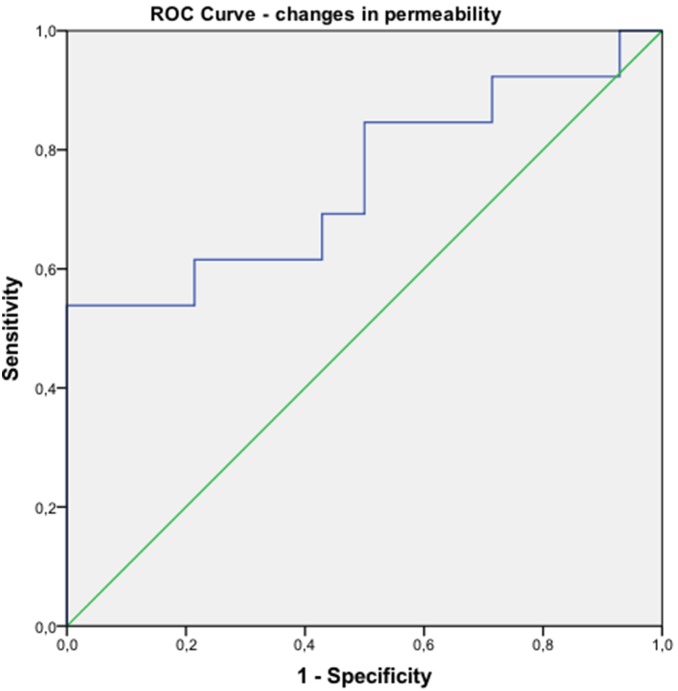
ROC analysis. ROC curve with a cut-off value of 25% reduction in permeability between baseline and early follow up scan. Area under curve is 0.74 (Confidence interval 0.55–0.93).

Using ROC analysis (Figure 5), a cut-off value of more than 25% reduction in tumour permeability yielded a sensitivity of 69% and a specificity of 58% for predicting response after the first series of chemotherapy.

### Clinical Response: Changes in Perfusion Parameters and Tumour Volume between Baseline and following 3 Series of Chemotherapy

After three series of chemotherapy, the probability of responding was correlated with a decrease in tumour permeability (p = 0.03). A decrease in tumour volume was also positively correlated with response (p = 0.01). The absolute change in blood volume was positively correlated with response (p = 0.03), but not in percentage change (p = 0.08).

Table 3 summarises changes in perfusion parameters and tumour volume after the first series of chemotherapy and after three series of chemotherapy.

**Table 3:** Changes in perfusion parameters and tumour volume during preoperative chemotherapy.

	**Clinical Nonresponders**			**Clinical Responders**			**Statistics** *			
	**Baseline**	**1. series**	**3. series**	**Baseline**	**1. series**	**3. series**	**After 1st series**		**After 3rd series**	
							**OR**	**p-value**	**OR**	**p-value**
**Arterial flow**	107.4	91.6	102.3	116.1	100.2	88,1	0.992	p = 0.35	0.990	p = 0.31
mL⋅min^−1^⋅100g^−1^	[96.5; 146.0]	[86.5; 148.4]	[81.1; 121.8]	[84.5; 140.8]	[75.5; 125.9]	[61.0; 103.6]	CI (0.973–1.010)		CI (0.972–1.009)	
		*−7%*	*−12%*		*−9%*	*−25%*	0.982	p = 0.23	0.986	p = 0.32
		*[−13%; 22%]*	*[−27%; 7%]*		*[−20%; 0%]*	*[−30%; −11%]*	CI (0.953–1.012)		CI (0.959–1.014)	
**Blood volume**	6.7	5.1	5.0	6.3	5.5	3.4	0.886	p = 0.39	0.699	**p = 0.03**
mL⋅100g^−1^	[4.1; 7.5]	[4.0; 6.7]	[3.7; 8.0]	[3.4; 9.6]	[3.5; 6.6]	[2.5; 5.2]	CI (0.740–1.125)		CI (0.506–0.966)	
		*−9%*	*−15%*		*−27%*	*−47%*	1.002	p = 0.56	0.982	p = 0.08
		*[−34%; 21%]*	*[−49%; 20%]*		*[−41%; −3%]*	*[−10%; −67%]*	CI (0.995–1.010)		CI (0.962–1.002)	
**Permeability** (k^trans^)	25.2	28.4	26.9	28.9	20.1	16.3	0.880	p = **0.03**	0.881	**p = 0.03**
mL⋅min^−1^⋅100g^−1^	[20.9; 34.9]	[19.8; 38.9]	[18.3; 32.4]	[23.0; 30.3]	[15.5; 23.8]	[11.3; 20.1]	CI (0.786–0.985)		CI (0.786–0.987)	
		*+2%*	*+1%*		*−30%*	*−43%*	0.964	p = **0.03**	0.962	**p = 0.03**
		*[−31%; 37%]*	*[−30%; 18%]*		*[−41%; −3%]*	*[−53%; −16%]*	CI (0.933–0.996)		CI (0.930–0.996)	
**Tumour volume**	34.3	38.9 **	38.8	30.8	21.8	11.4	0.987	p = 0.63	0.916	p = **0.03**
mL	[22.7; 59.9]	[21.0; 54.2]	[14.7; 54.7]	[9.8; 51.6]	[7.1; 38.3]	[5.5; 23.0]	CI (0.947–1.033)		CI (0.846–0.991)	
		*−14%*	*−10%*		*−29%*	*−56%*	0.992	p = 0.47	0.947	p = **0.01**
		*[−37%; 13%]*	*[−33%; 18%]*		*[−43%; −1%]*	*[−73%; −47%]*	CI (0.970–1.014)		CI (0.910–0.987)	

Perfusion parameters and tumour volume before, after first series of chemotherapy and after third series of chemotherapy grouped by clinical response. Reported values are median values and interquartile range.

*Odds ratio and 95% confidence intervals are reported on both absolute changes and changes in percentage using logistic regression. Significant p-values are highlighted.

**Although the median is higher after first series of chemotherapy versus baseline, the median change is negative.

### Histological Response: Changes in Perfusion Parameters and Tumour Volume during Treatment

**Figure 6:**
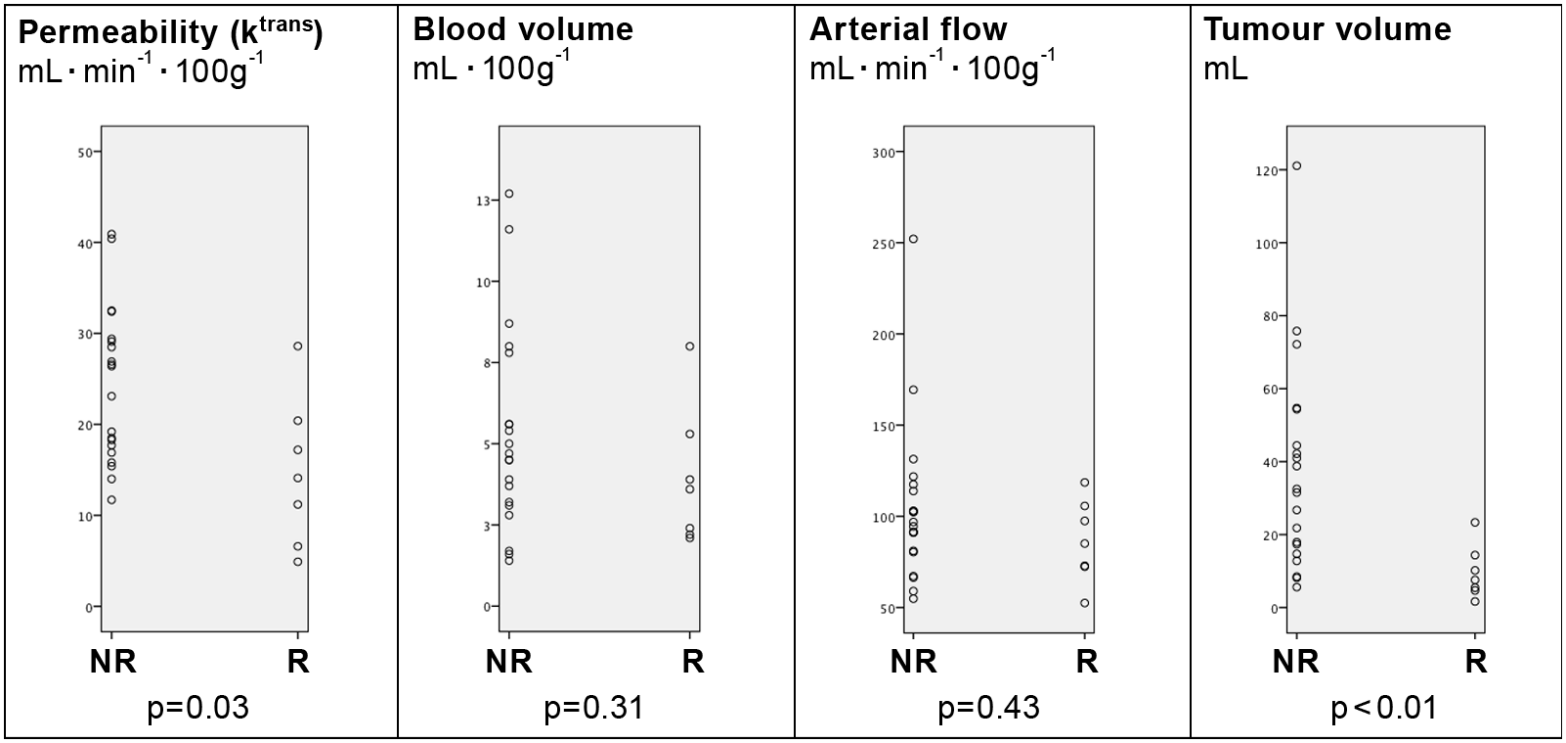
Pre-operative scan and histological response based on Mandard Score. Correlation between perfusion parameters pre-operatively and histological response. n = 27.

The logistic regression analysis showed that the probability of a histological response increased with a reduction in permeability (p = 0.03) and in tumour volume (p = 0.03) between baseline scan and after third series of chemotherapy. Changes in arterial flow and blood volume were not significant (p = 0.39 and p = 0.89, respectively). We observed no significant changes after the first series of chemotherapy in arterial flow (p = 0.39), blood volume (p = 0.42), permeability (p = 0.20), or tumour volume (p = 0.25). Comparison of perfusion parameters and tumour volume of the preoperative scan with histological response showed a significant difference in permeability between histological responders and nonresponders (p<0.05), as demonstrated by clinical response. Likewise, responding patients had significantly smaller tumours than non-responding patients (p<0.01). There were no statistically significant differences in blood volume (p = 0.31) and arterial flow (p = 0.43) (Figure 6).

### One-year Follow up for Recurrence of Disease

Within the first year after inclusion 8 out of the 27 resected patients (30%) had recurrence of disease. Four of these 8 were clinical responders after surgery (4 out of all 13 clinical responders = 31%) and 1 was a histological responder (1 out of all 8 histological responders = 13%).

## Discussion

The main finding of this study was that there was a positive correlation between a decrease in tumour permeability (k^trans^) after one series of chemotherapy (three weeks) and treatment response after three series of chemotherapy measured by clinical response. This correlation appeared before significant changes in tumour volume were detectable in the scans. Patients with a histological response had a significantly lower tumour permeability compared to nonresponding patients. To our knowledge, this is the first longitudinal study using CT perfusion as a method to measure early response to pre-operative chemotherapy in GEJ and gastric cancer.

Perfusion is the delivery of blood through the vascular bed and can be estimated using different kinetic models. The basis of CT perfusion is the transport of a contrast material by blood flow through the intravascular space, and subsequently, the distribution of the contrast material between the intravascular and extravascular interstitial space [20],[21]. Previous studies using CT perfusion in esophageal and gastric cancer have found a significant correlation with blood volume [22]–[24] and permeability surface area product [22], and histological evaluation of micro vessel density [22]–[24] and Vascular Endothelial Growth Factor (VEGF) expression [25]. Likewise, Satoh et al. [26] found a negative correlation between tumour perfusion and stromal density. Makari et al. [27] examined changes in tumour perfusion before and after chemo-radiation in esophageal squamous cell carcinoma and found a positive correlation between perfusion changes and tumour size reduction. Our baseline perfusion measures are in the same range as some studies [22], [28], and differs from other studies [24], [25]. The analysis software used in this study is based on two kinetic models: the maximum slope method to estimate tissue perfusion and Patlak method to estimate both blood volume and permeability (k^trans^), which is the rate constant for transfer between the intravascular and extravascular (interstitial) spaces. The lack of standardisation of CT perfusion protocols, and implementation of kinetic models complicate direct comparison of values between studies.

The first adoptions of the CT perfusion technique were limited to single slice studies. With the use of a 320-detector row CT scanner it is possible to cover 16 cm in a single gantry rotation, thereby improving the temporal resolution compared to a helical scan [29]. A 16 cm coverage enables correction for motion in all three planes, which allows a free-breathing scan protocol, and makes three-dimensional volume perfusion measurements possible [30]. We observed a significant correlation between a reduction in permeability and response to chemotherapy. It has been shown, that cisplatin inhibits the expression of growth factors e.g. in ovarian cancer cells [31], [32] and as growth factors are important for angiogenesis in tumour growth, this inhibition could explain some of the observed reductions in permeability. Previous longitudinal studies using CT perfusion of colorectal cancer [7], [8] have demonstrated a significant post-therapeutic reduction in blood volume (84 and 91 days, respectively), but these studies did not examine any differences in changes between responders and nonresponders. A post-therapeutic reduction in blood volume has been demonstrated in lung cancer [33], [34], in blood flow in liver metastasis after 3 weeks [4], [35] and in blood volume in animal models of hepatic tumours [36], [37]. Our study did not show any early changes in blood volume, but the reduction was significant in absolute numbers after three series of chemotherapy (table 3).

The clinical response rate in our study was 46% (13/28), which is comparable to those of other response evaluation studies of esophageal and gastric cancer [15]. Not all patients exhibiting a clinical response also demonstrated a histological response. Despite a significant reduction in tumour volume, some regions of tumour might display groups of viable tumour cells, which would classify the patient as a non-responder based on histopathological grading. Histopathological evaluation of regression is considered a gold standard for response evaluation and is clearly associated with survival (34). In our study, only 1 out of 8 patients with histological response had recurrence within the first year, whereas 4 out of 13 patients with clinical response had recurrence. This indicates, that response evaluation based on tumour size reduction is unreliable. The clinical utility of histopathological grading is however limited to modifying only the postoperative, adjuvant therapy.

Other imaging modalities have been used for response assessment of preoperative chemotherapy for adenocarcinomas in the esophagus and at the gastroesophageal junction. Studies using Positron Emission Tomography (PET) with fludeoxyglucose (FDG) as tracer [13], [14] have demonstrated a larger reduction in FDG uptake in responding versus nonresponding tumours after 14 days of neoadjuvant chemotherapy. The MUNICON-1 trial [38] indicated that chemotherapy can be discontinued at an early stage in metabolic nonresponders without compromising the prognosis. MUNICON-2 [39] showed that the addition of neoadjuvant radiotherapy in metabolic nonresponders did not lead to an improvement of their poor prognosis, thus showing that early non-response indicates dismal tumour biology. The MUNICON trials thus illustrated the feasibility of a PET-response-guided treatment algorithm. However, larger, randomised trials are needed. A large fraction of gastric cancers are also found to be non-FDG-avid and PET has been proven not as informative in the evaluation of early response to chemotherapy [40], [41]. The advantages of CT perfusion compared to PET-FDG are the great availability of scanners, the easy handling of contrast material, and the possibility of adding vascular physiological measures to already existing diagnostic protocols. CT perfusion could also be applied in a combination with PET/CT imaging.

There are some limitations to our study. First, our study sample is small and the data presented should be interpreted with this in mind. Secondly, albeit GEJ and gastric cancer arise from the same epithelial cells, they are located at different sites in the gastrointestinal tract, with possible different outcome to treatment. There is consensus on treating the cancers in the same peri-operative setting, and the MAGIC trial [10] and a study by Ychou et al. [9] confirms no difference in outcome between esophageal and gastric cancer. Thirdly, cancers in the gastrointestinal tract are difficult tumours to visualise with high resolution imaging technology. The organs are affected both extrinsically by respiratory and cardiac movement, and intrinsically due to peristalsis and varying distension of the lumen. We compensated for some of these factors by applying motion correction to the dataset and administering a motility inhibitor. Fourthly we included all patients with both GEJ and gastric cancer eligible for perioperative chemotherapy and made no selection bias on tumour size. The tumour sizes in our study were smaller compared to other studies with response evaluation [13], [38]. Lastly, our clinical response evaluation is based on tumour size reduction. There is evidence of histological response evaluation as predictor of recurrence and overall survival, whereas validation on tumour size reduction (clinical response) lacks. Our 1-year follow up indicates a higher frequency of recurrence among clinical responders compared to histological responders.

In conclusion, our study found a positive correlation between an early decrease in tumour permeability (k^trans^) after one series of chemotherapy and the response after three series of chemotherapy, based on tumour size reduction. A cut-off value with a reduction in permeability of more than 25% gives a sensitivity of 69% and a specificity of 58% after one series of chemotherapy, which makes the diagnostic quality moderate and insufficient as a single discriminatory test. With regards to histological response, our study finds significantly lower permeability in responding tumours compared to non-responding after three series of chemotherapy, but not after the first series of chemotherapy. Histological response evaluation seems better than clinical response to estimate the risk of recurrence of disease.

## Supporting Information

Checklist S1 **The REMARK checklist.** **Study details in accordance with reporting recommendations for tumor marker prognostic studies (REMARK).**
(DOC)
