# Supplementary material for: Computed Tomography (CT) Perfusion as an Early Predictive Marker for Treatment Response to Neoadjuvant Chemotherapy in Gastroesophageal Junction Cancer and Gastric Cancer - A Prospective Study
Source: PLoS One. 2014 May 20;9(5):e97605. doi: 10.1371/journal.pone.0097605 (PMC4028233; doi:10.1371/journal.pone.0097605)
Supplement: Checklist S1 — The REMARK checklist. Study details in accordance with reporting recommendations for tumor marker prognostic studies (REMARK). (DOC) [file pone.0097605.s001.doc]

| **Item to be reported** | | **Section** |
| --- | --- | --- |
| **INTRODUCTION** | |  |
| 1 | *Marker, objectives and hypothesis* Computed Tomography (CT) Perfusion derived physiological parameters – tissue perfusion, blood volume and permeability. With this study we wanted to evaluate whether an early reduction in perfusion parameters could predict response to pre-operative chemotherapy. Our hypothesis being that chemotherapy induces changes in tumour and an early reduction in perfusion parameters could predict response to pre-operative chemotherapy | introduction |
| **MATERIALS AND METHODS** | |  |
| *Patients* | |  |
| 2 | *Characteristics* Thirty consecutive patients with biopsy proven adenocarcinoma at the gastro-esophageal junction or in the stomach were included. *Inclusion criteria* Tumour visible on diagnostic CT scan, considered potentially resectable at a multidisciplinary tumour (MDT) conference.  *Exclusion criteria* Contrast allergy, impaired renal function, and patients unfit for chemotherapy. | Patients |
| 3 | *Treatment* Three cycles of chemotherapy preoperatively. Each 3-week cycle consisted of epirubicin and cisplatin / oxaliplatin intravenously on day 1 combined with peroral capecitabine continuously for 21 days. Hereafter surgery. | Treatment |
| *Specimen characteristics* | |  |
| 4 | Formalin-fixed paraffin-embedding tumour tissue. Hematoxylin and eosin stain. | Response evaluation |
| *Assay methods* | |  |
| 5 | CT Perfusion scan using a 320-detector row scanner with a scan-field of 12 – 16 cm. The protocol consisted of 19 consecutive volumes with variable start determined with a test-bolus. Contrast volume ranged from 30 to 40 mL. Image acquisition with the following parameters: 100 kV, 100 mA, 0.5 s/rotation time, fixed table position, and 0.5 mm reconstruction. Image reconstruction with iterative reconstruction and a non-rigid motion correction algorithm. Image analysis in consensus by two readers. | Perfusion CT |
| *Study design* | |  |
| 6 | Prospective inclusion with 3 perfusion scans. Baseline (median 8 days before starting chemotherapy, range 3-37 days), after the first cycle of chemotherapy (median 20 days, range 18-31 days) and before surgery (median 79 days, range 61 – 102 days). | Perfusion CT |
| 7 | *Clinical endpoints* Macroscopic tumour size, histological regression score evaluation (Mandard score) | Response evaluation |
| 8 | *Variables considered at start* Age, tumour size, tumour stage, weight, dysphagia. | Patients |
| 9 | *Study planning* The trial was planned to include 30 patients prospectively. |  |
| *Statistical analysis methods* | |  |
| 10 | Changes in perfusion between scans were compared on a group basis by response using univariate logistic regression. Perfusion parameters at baseline and at the third scan were compared between responders and nonresponders using Mann Whitney U test. Comparison between tumour tissue and normal gastric tissue was done using a Wilcoxon signed-rank test. A 2-sided p-value below 0.05 was considered statistically significant. A Reciever Operating Characteristic (ROC) analysis was made to determine the optimal diagnostic cut–off value for changes in perfusion parameters. | Statistics |
| 11 | Changes in perfusion parameters were analysed as changes in percentage and absolute numbers. | Statistics |
| **RESULTS** | |  |
| *Data* | |  |
| 12 | **Patient flow** Thirty patients were included. One patient only completed the first scan and one patient was excluded due to motion artefacts on all three perfusion scans. The remaining 28 patients constituted the study group (24 male, 4 female, median age 65, range 44 - 79). Twenty-six patients completed all three perfusion scans. One patient was excluded from the third scan due to elevated serum creatinine levels, and one patient missed the second scan due to hardware failure, leaving 27 patients available for early response evaluation and 27 for presurgical response evaluation. | Patients |
| 13 | 28 cases were available for follow-up analysis (24 male and 4 female, median age 65, range 44 - 79). See Table 2 for further characteristics. | Patients |
| *Analysis and presentation* | |  |
| 14 | Data analysis is presented graphically in figure 1, 3 and 4 with changes in perfusion parameters grouped by tumour size reduction (clinical response as outcome).   Statistical tests on each perfusion variable is presented in table 3 with clinical response as outcome and odds ratio for response.  Figure 5 illustrates correlation between perfusion parameters after three series of chemotherapy and histological response according to the Mandard Score.  One-year follow up after inclusion showed that 8 out of the 27 resected patients (30%) had recurrence of disease. Four of these 8 were clinical responders after surgery (4 out of all 13 clinical responders = 31%) and 1 was histological responder (1 out of all 8 histological responders = 13%) |  |
| 15 |  | Results |
| 16 |  |  |
| 17 |  |  |
| 18 |  |  |
| **DISCUSSION** | |  |
| 19 | **Interpret the results in the context of the pre-specified hypotheses and other relevant studies; include a discussion of limitations of the study.**  Our study finds a positive correlation between a decrease in tumour permeability (k^trans^) after one series of chemotherapy (three weeks) and the odds of clinical response after three series of chemotherapy (twelve weeks). A cut-off value with a reduction in permeability of more than 25% gives a sensitivity of 69% and a specificity of 58%, which makes it insufficient as a single discriminatory test. In regards to histological response, our study finds significantly lower permeability in responding tumours compared to non-responding after three series of chemotherapy, but not after the first series of chemotherapy. | Discussion |
| 20 | As a single diagnostic setup, CT perfusion has moderate sensitivity and specificity. | Conclusion |
